# Liver Injury Following Intravenous Methylprednisolone Pulse Therapy in Multiple Sclerosis: The Experience from a Single Academic Liver Center

**DOI:** 10.3390/biom15030437

**Published:** 2025-03-19

**Authors:** Dimitris Kounatidis, Natalia G. Vallianou, Georgios Kontos, Hariklia Kranidioti, Nikolaos Papadopoulos, Alexandros Panagiotopoulos, Krystalia Dimitriou, Vasileios Papadimitropoulos, Melanie Deutsch, Spilios Manolakopoulos, Dimitrios Vassilopoulos, John Koskinas

**Affiliations:** 1 Second Department of Internal Medicine, Medical School, National and Kapodistrian University of Athens, Hippokration General Hospital, 11527 Athens, Greece; dimitriskounatidis82@outlook.com (D.K.); dr.gkontos@gmail.com (G.K.); harakranidioti@yahoo.gr (H.K.); krystalia_dim@hotmail.com (K.D.); bpapad@hotmail.com (V.P.); meladeut@gmail.com (M.D.); smanolak@med.uoa.gr (S.M.); dvassilop@med.uoa.gr (D.V.); koskinasj@yahoo.com (J.K.); 2 Diabetes Center, First Department of Propaedeutic Internal Medicine, Medical School, National and Kapodistrian University of Athens, Laiko General Hospital, 11527 Athens, Greece; 3 First Department of Internal Medicine, Sismanogleio General Hospital, 15126 Athens, Greece; 4 Second Department of Internal Medicine, 401 General Army Hospital of Athens, 15561 Athens, Greece; nipapmed@gmail.com; 5 Rheumatology Unit, First Department of Propaedeutic Internal Medicine, Joint Academic Rheumatology Program, Medical School, National & Kapodistrian University of Athens, Laiko General Hospital, 11527 Athens, Greece; alej.panagiotopoulos@gmail.com

**Keywords:** autoimmune hepatitis, drug-induced autoimmune-like hepatitis, drug-induced liver injury, hepatotoxicity, methylprednisolone, multiple sclerosis

## Abstract

Intravenous methylprednisolone (IVMP) pulses, widely used for managing multiple sclerosis (MS) exacerbations, can lead to acute liver injury, presenting a diagnostic challenge in distinguishing between drug-induced autoimmune-like hepatitis (DI-ALH) and idiopathic autoimmune hepatitis (AIH). This study aimed to delineate the clinical and biochemical features of IVMP-induced liver injury, discern its etiology, and evaluate the efficacy of glucocorticoid (GC) therapy in treatment. A retrospective analysis of 13 relapsing MS patients with IVMP-induced liver injury was conducted. Liver injury was classified as hepatocellular, cholestatic, or mixed, with severity assessment guiding liver biopsy in selected cases. Causality was assessed using the Roussel Uclaf Causality Assessment Method (RUCAM) and the Simplified Diagnostic Criteria for AIH. All patients were initially monitored for a minimum of six months, with a mean follow-up period of 4.30 years. The median onset of liver injury was 37.46 days post-IVMP, with a mean peak alanine transaminase (ALT) level of 618.46 U/L. antinuclear antibody (ANA) positivity was observed in 61.53% of cases, with elevated serum immunoglobulin G (IgG) at 15.38%. Hepatocellular injury was universal among patients, and causality assessment predominantly supported DI-ALH. GC therapy was administered in six cases, achieving favorable outcomes in all but one, which necessitated rituximab. Biochemical normalization occurred within a mean of 55.41 days, with GC-treated patients recovering faster (48 days). These findings support the hypothesis that IVMP can induce hepatocellular injury, likely DI-ALH, during MS exacerbations. A tapering GC regimen proved effective in promoting recovery, particularly in severe cases. Additionally, this study introduced a diagnostic and therapeutic algorithm for managing IVMP-induced liver injury, offering a practical framework for clinical application.

## 1. Introduction

Multiple sclerosis (MS) is a chronic autoimmune disorder of the central nervous system (CNS), marked by inflammation, demyelination, and neuronal damage [1]. High- dose intravenous methylprednisolone (IVMP) pulses have long been a cornerstone therapy for managing MS exacerbations [2,3]. However, while rare, IVMP administration can predispose subjects to liver injury, with certain risk factors heightening the likelihood of hepatotoxicity [4]. The clinical spectrum of IVMP-related liver injury ranges from asymptomatic elevations in liver enzymes to severe clinical manifestations, such as jaundice and encephalopathy, which, though infrequent, may prove life-threatening [5].

The majority of the existing literature suggests that IVMP-induced hepatitis is a phenotype of idiosyncratic drug-induced liver injury (DILI), which may present biochemical, immunological, and histological features resembling idiopathic autoimmune hepatitis (AIH) [6]. This clinical entity is often referred to as drug-induced autoimmune- like hepatitis (DI-ALH), a term also adopted in this manuscript [7]. Conversely, limited evidence indicates that IVMP infusion could potentially unmask latent AIH, considering that MS patients are at a tenfold greater risk of developing AIH compared to the general population [8,9].

Building on this background, we conducted a retrospective observational study of 13 patients monitored at our center over an 18-year period. These patients, all with a history of MS, developed hepatitis following IVMP administration during relapse management. Patient evaluation included a detailed medical history, physical examination, and laboratory investigations, encompassing viral hepatitis exclusion, serum immunoglobulin G (IgG) quantification, and immunological testing. Liver ultrasonography (US) was performed to rule out other causes of hepatic damage. Both the pattern and severity of liver injury were assessed, with liver biopsy conducted in hospitalized patients (two of three cases, as one patient declined). Causality was assessed using the Roussel Uclaf Causality Assessment Method (RUCAM) and the Simplified Diagnostic Criteria for AIH [10,11].

Demographic data, including age and gender, were analyzed alongside IVMP-related parameters, such as the latency to liver injury onset and the rate of liver biochemistry normalization, with or without glucocorticoid (GC) intervention. All patients were initially followed for a minimum of six months, with 10 out of 13 monitored for at least five years. The remaining three cases, which were clinically severe, had a shorter follow-up period of two years due to their recent diagnosis. Finally, by integrating our study findings with the existing literature, we developed a diagnostic and therapeutic algorithm to guide the management of this challenging clinical entity.

## 2. Materials and Methods

### 2.1. Patient Population

We performed a retrospective analysis of 13 individuals who encountered hepatitis following IVMP pulse treatment for relapsing MS between May 2006 and November 2024. The study was conducted in accordance with the Declaration of Helsinki for research involving human subjects. Ethical approval was obtained from the Ethical Committee of Hippokration General Hospital under protocol number 79/26-02-2025, date: 26 February 2025. Each participant received 1 g of IVMP over a period of 2 to 5 days. Among them, five subjects were undergoing disease-modifying therapy (DMT), while six had a documented history of prior IVMP pulse treatment. Notably, all but one individual had no prior diagnosis of other autoimmune diseases. The sole exception, patient 13, had a previous diagnosis of AIH, which was in remission and managed with mycophenolate mofetil (MMF). All participants had normal liver biochemistry prior to the administration of the IVMP pulses. A thorough collection of medical histories was performed, including serological screening for viral causes (hepatitis A, B, C, E, Epstein–Barr virus [EBV], Cytomegalovirus [CMV], Human Immunodeficiency Virus [HIV]). Moreover, serum IgG levels, along with autoimmune serological tests (antinuclear antibodies [ANA], antimitochondrial antibodies [AMA], anti-smooth muscle antibodies [ASMA], soluble liver antigen/liver–pancreas antibodies [SLA/LP], liver cytosol type 1 antibodies [LC], and liver–kidney microsomal antibodies [LKM]) were determined. Liver US imaging studies were also performed, while 2 individuals with clinically significant liver injury underwent liver biopsy. Furthermore, we assessed the latency period following IVMP injection and identified the interval between methylprednisolone (MP) administration and the normalization of liver function. Additionally, we recorded the percentage of patients who were treated with GCs and compared the rate of liver biochemistry normalization between those who received GC therapy and those who did not.

### 2.2. Definition of the Type of Liver Injury

The type of acute liver injury was determined using the ratio (R) of baseline alanine aminotransferase (ALT) elevation to baseline alkaline phosphatase (ALP) elevation, calculated as (ALT/ALT_ULN)/(ALP/ALP_ULN), where ULN stands for upper limit of normal. Acute hepatocellular injury was classified by an ALT increase >5× ULN or an R > 5. Conversely, acute cholestatic liver injury was defined as an ALP increase >2× ULN or an R < 2. Acute mixed liver injury was identified by an R between 2 and 5 [12].

### 2.3. Definition of the Severity of Liver Injury

The assessment of DILI severity relies on an integration of clinical presentation and laboratory parameters. Patients were initially classified into categories of non-severe and severe liver injury. Clinically significant DILI was defined by the fulfillment of at least one of the following criteria: 1. Serum AST or ALT levels exceeding 5 times the ULN, or ALP levels surpassing 2 times the ULN (or the baseline value if baseline is elevated) on two separate occasions; 2. Total serum bilirubin concentration exceeding 2.5 mg/dL along with elevated AST, ALT, or ALP; 3. International normalized ratio (INR) exceeding 1.5 in conjunction with elevated AST, ALT, or ALP. Furthermore, patients were assessed for the presence of jaundice, encephalopathy, or multiorgan failure [13].

### 2.4. Causality Evaluation for DILI Diagnosis

Various scoring systems have been developed to aid in the diagnosis of DILI, including the RUCAM [14]. This method evaluates agents with potential hepatotoxicity across multiple categories, generating a score that classifies the likelihood of a drug being the cause of liver injury as highly probable (>8), probable (6–8), possible (3–5), unlikely (1–2), or excluded (<0). Consistent with Allgeier, in the “known hepatotoxicity” category, one point was assigned for MP. This allocation is justified by the fact that liver injury associated with MP has been documented solely in the literature and is not yet incorporated into the drug labeling process [15].

### 2.5. Autoimmune Hepatitis Score Calculation

As liver biopsy was not performed in most cases, we opted to compute AIH scores utilizing the Simplified Diagnostic Criteria for Autoimmune Hepatitis. A score below 6 generally rules out the diagnosis of AIH. Conversely, a score equal to or exceeding 6 is considered indicative of probable AIH, while a score surpassing 7 is indicative of definite AIH [11]. Liver biopsy was conducted in two patients with clinically significant liver injury. Therefore, we calculated the maximum score based on screening findings for viral hepatitis, immunological tests, and serum IgG levels. In situations where a score is less than 3, AIH is typically improbable. Thus, even with 2 points added from the liver biopsy, the maximum score does not exceed 5.

## 3. Results

Most subjects were female (12/13), with a median age of 40.07 years (25–60 range). The mean latency period was 37.46 days, ranging from 15 to 75 days. Upon admission, the mean ALT value was 539.61 U/L (ranging from 93–2318 U/L). In addition, the mean peak ALT value during the monitoring period was 618.46 U/L (97–2318 U/L range). In all cases, hepatocellular liver injury was observed. Viral hepatitis was excluded in all participants, serum IgG was increased in 2 cases (ULN 1600 mg/dL), and positive ANA were detected in 61.53% of the patients (8/13).

Liver US showed no evidence of advanced chronic liver disease, while three patients experienced severe liver damage. Liver biopsy was performed in 2 out of 3 of these cases (cases 2 and 13), as one patient (case 3) declined. In both cases, the biopsy revealed portal hepatis. In case 2, histological examination showed infiltration by neutrophils and lymphocytes, whereas case 13 exhibited centrilobular necrosis. The mean RUCAM was 7 (5–10).

All subjects were evaluated for treatment outcomes. Seven participants (53.85%) with non-severe liver injury did not receive specific treatment, and all experienced remission within an average of 60.71 days (spanning from 1 to 3 months). Conversely, GCs were prescribed to 6 out of 13 patients (46.15%), including the 3 patients with severe liver injury. Prednisolone at a daily dose of 40 mg was the most frequently used treatment, while one patient (case 11) was treated with budesonide. In 5 of the 6 cases, a 3-month GC-tapering regimen resulted in favorable outcomes, as observed during an initial 6-month follow-up period, with normalization of liver enzymes occurring at an average of 48 days. Unlike the other patients, patient 13, with a prior history of AIH in remission, did not respond to GC therapy but showed a positive response to salvage treatment with rituximab.

Over a five-year follow-up, 10 of the 13 participants showed no recurrence of liver injury. The remaining three patients, all with severe liver damage and relatively recent diagnoses, did not experience recurrence within a two-year follow-up period. None of the patients underwent rechallenge, although six had a history of previous IVMP pulses. Notably, patient 3 had experienced a similar acute liver failure episode five years prior, which was resolved spontaneously without treatment. Patient 11 had also received steroid pulses on two prior occasions without developing hepatitis. In the current episode, GCs followed a 12-day tapering regimen after MP pulses, while previous episodes had involved monthly MP tapering.

Table 1 provides the main demographic, clinical, and laboratory data, while the analytical characteristics of the patients are detailed in Supplementary Tables S1 and S2.

## 4. Discussion

MS is a chronic, progressive, and demyelinating disorder of the CNS, characterized by unpredictable relapses and remissions. Over the years, the therapeutic landscape for MS has evolved significantly, primarily with the introduction of DMTs. Despite these advancements, high-dose IVMP pulses, typically administered at doses ranging from 500 mg to 1 g daily for 3 to 5 days, remain the conventional treatment for acute MS exacerbations [16]. In recent years, attention has been drawn to IVMP-induced liver injury as an emerging and distinct complication in this patient population [17]. This adverse effect has also been reported in other autoimmune disorders, particularly in Graves’ orbitopathy (GO) [18].

Several predisposing factors have been identified in the development of IVMP- induced hepatitis. These include hyperlipidemia, alcohol abuse, and metabolic- associated fatty liver disease (MAFLD) [19]. Despite limited data, current evidence indicates that the incidence of hepatotoxicity following the administration of IVMP pulses in MS patients ranges from 2.86% to 8.6%. These data are derived from three studies that exclusively investigated liver injury in this specific population [19,20,21]. Conversely, other studies report significantly higher incidence rates, with some reaching 25.9% [19,20,22,23]. This variability can be attributed to differences in the criteria used to define liver injury, diverse methodologies for causality assessment, and variations in follow-up protocols across studies.

For instance, Ueno et al. documented an incidence of hepatitis in only 1.2% of patients receiving IVMP. It is important to note that this study applied stringent exclusion criteria, such as omitting patients who experienced spontaneous remission of liver injury without undergoing histological evaluation [24]. Furthermore, unlike other studies that defined liver injury solely based on ALT levels exceeding 45 IU/L [19,20], Ueno et al. adopted a more comprehensive diagnostic approach, incorporating ALT, ALP, and TBIL levels [24]. An intriguing aspect of their research is that all five patients ultimately classified as having IVMP-induced hepatitis had underlying neurological diseases, with four out of five being women [24]. This female predominance aligns with a literature review of 50 published cases of IVMP-induced hepatotoxicity, which revealed that 86% of cases involved female patients. Among these cases, 29 were individuals diagnosed with MS [25]. Similar trends were observed in our study and in the work of Allgeier et al., which featured a comparable sample size and methodology. Both studies reported a mean age of onset for IVMP-induced hepatitis of approximately 40 years [15].

On the other hand, the study by Eguchi et al., which focused on patients with GO, highlighted the influence of additional factors such as the severity of liver injury, patient age, and cumulative IVMP dose on gender-specific risks. According to this study, men were at higher risk of developing mild liver dysfunction when the cumulative IVMP dose exceeded 8 g, whereas women over 50 years of age were more likely to develop moderate liver dysfunction [26]. Interestingly, the study revealed a dose-dependent association between IVMP doses exceeding 8 g and hepatotoxicity. Additionally, other data suggest that an increased cumulative IVMP dose may also be linked to higher mortality rates [25]. These findings are in contrast with the typically dose-independent nature of idiosyncratic DILI [27].

The predominant hypothesis suggests that IVMP pulses induce transient immunosuppression, followed by a rebound phenomenon upon cessation, resembling immune reconstitution inflammatory syndrome (IRIS). IRIS is typically observed in HIV/acquired immunodeficiency syndrome (AIDS) patients during the initial months of highly active antiretroviral therapy (HAART), where it is believed to result from an exaggerated immune response to opportunistic pathogens as CD4^+^ T cell counts recover [28]. In contrast, the exact mechanism underlying IVMP-induced hepatitis remains unclear, although some authors propose that the abrupt discontinuation of IVMP therapy may uncover latent AIH in predisposed individuals [25]. Interestingly, the current literature suggests that in non-infectious causes of IRIS, autoimmunity to innate antigens presumably plays a role in syndrome development [29].

One compelling theory that may partially explain the pathophysiological changes involved focuses on the immunomodulatory effects of GCs, particularly their ability to suppress pro-inflammatory mediators that initiate the inflammatory cascade. During the early phases of IVMP therapy, the production of pro-inflammatory cytokines such as tumor necrosis factor-alpha (TNF-α), interferon-gamma (IFN-γ), and interleukin-17 (IL-17) is significantly inhibited [30,31]. However, upon the abrupt withdrawal of IVMP, these cytokines undergo a rapid surge, triggering a hyper-inflammatory state (a hallmark of IRIS in HIV/AIDS patients) that leads to hepatic injury [32,33]. An underlying autoimmune condition appears to be a prerequisite for this phenomenon, although no genetic predisposition has been identified thus far [25]. Supporting this theory is the observation that the risk of hepatic damage is reduced when discontinuation of IVMP is followed by GC administration, specifically with prednisolone [34]. Nonetheless, high-quality studies are necessary to fully clarify the mechanisms involved.

**Figure 1:**
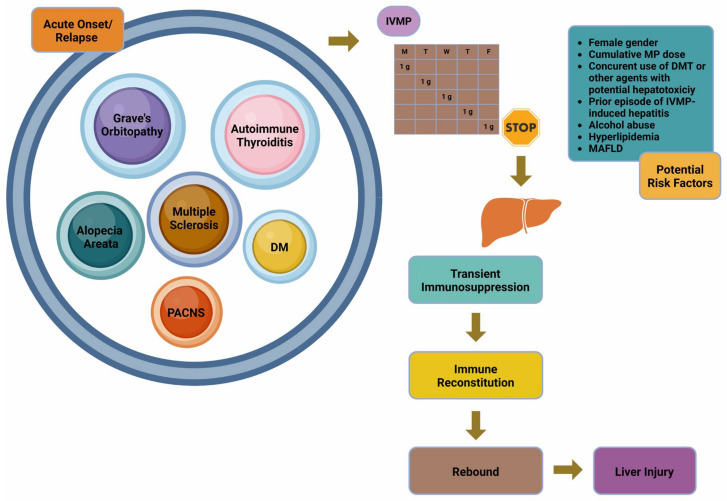
Primary causes, risk factors, and pathophysiology of IVMP-induced hepatitis: This figure illustrates the main causes and potential risk factors associated with IVMP-induced hepatitis. It also depicts the prevailing hypothesis regarding the pathogenetic mechanism underlying this condition. Abbreviations: DM: dermatomyositis; DMT: disease-modifying treatment; IVMP: intravenous methylprednisolone; MAFLD: metabolic-associated fatty liver disease; MP: methylprednisolone; PACNS: primary angiitis of the central nervous system. Created in BioRender. Kounatidis, D. (2025) https://BioRender.com/q18r153, accessed on 1 March 2025.

Figure 1 provides an overview of the primary causes, risk factors, and proposed pathophysiological mechanism of IVMP-induced hepatitis.

A pertinent question that arises is why this phenomenon occurs specifically with MP and not with other classes of GCs. Currently, there is a lack of studies directly comparing different GC classes in this context. A plausible explanation is that IVMP pulses represent the standard treatment regimen in MS, unlike other GCs such as prednisolone, which is not routinely used in this context [2,3]. On the other hand, although data remain limited and are primarily derived from case reports, dexamethasone appears to be a safe therapeutic alternative for managing MS relapses without any reported cases of liver injury. Dexamethasone has been administered in various three-day therapeutic regimens, ranging from 50 to 96 mg per day [35,36]. Even at these relatively high doses (40 mg of dexamethasone is the typical pulse regimen in routine clinical practice) this does not equate to the 1 g of methylprednisolone usually administered during MS exacerbations [37]. Furthermore, given the scarcity of reports concerning dexamethasone, definitive conclusions regarding the comparative hepatotoxic risk of different glucocorticoid classes remain elusive.

The onset of liver injury associated with MP administration typically occurs more than two weeks after complete cessation of treatment, although delayed cases of recovery have been documented, extending up to seven months [21]. In accordance with these data, all patients in our study exhibited acute liver injury after 15 days post-MP pulse discontinuation. The longest latency period was observed in patient 5, reaching 75 days. The mean latency period for hepatic injury in our study was 37.46 days, closely aligning with the study by Allgeier et al., where the reported mean latency was 34 days [15]. Nevertheless, evidence also suggests that the latency period may be shorter, with cases of liver injury occurring as early as 10 days post-treatment [24].

Hepatocellular injury patterns are most common in patients with IVMP-related hepatitis most commonly present, as demonstrated in the majority of studies, including ours. However, rare cases of cholestatic or mixed injury patterns have also been documented [25]. The severity of liver injury remains unclear due to conflicting data, and direct comparisons across studies are challenging due to differences in methodologies, as previously mentioned. In our study, 23.07% of patients developed clinically significant liver injury, while other data suggest even higher incidence rates [25].

The diagnostic process for IVMP-induced hepatitis in MS patients is complex, primarily due to the difficulty in distinguishing DI-ALH from the potential onset of underlying idiopathic AIH. The process typically begins with a detailed review of the patient’s medical history to identify predisposing risk factors. Clinical evaluation offers limited diagnostic value, as the signs and symptoms of liver injury are often non-specific or even absent, tending to vary according to the severity of liver damage. Laboratory tests are essential to rule out other potential causes, such as viral hepatitis, and to assess the type and severity of liver injury. Subsequent steps involve measuring serum IgG levels and conducting immunological testing, with a range of autoimmune markers being assessed, including ANA, AMA, ASMA, SLA/LP, LC, and LKM-1/LKM-3, all of which are necessary for identifying the underlying cause of liver injury [38].

However, interpreting serum IgG levels and immunological test results can present significant challenges, since in patients with AIH, approximately 10–20% may exhibit normal IgG levels, particularly during the acute phase [39]. Furthermore, the presence of positive autoantibodies is not exclusive to AIH, as similar autoimmune profiles are also seen in DI-ALH [40]. Additionally, ANA may be found in the general population, especially in women, and many MS patients test positive for these autoantibodies despite the absence of clinically evident autoimmune disease [41,42]. On the other hand, MS patients have a ten-fold increased risk of developing AIH, with recent studies suggesting a potential role for human leukocyte antigen (HLA) expression in this association [43,44,45]. In our study, only two patients demonstrated elevated serum IgG levels, while eight out of thirteen patients tested positive for ANA. It is noteworthy that three patients (cases 6, 9, and 13) had positive ANA results in prior testing. In case 13, with a known history of AIH, the presence of ANA is expected. However, in cases 6 and 9, the immunological assays were conducted at external laboratories, precluding direct comparisons and limiting the reliability of definitive conclusions.

As anticipated, imaging techniques are not directly helpful in diagnosing DI-ALH or AIH. However, liver US is crucial for excluding other conditions, such as MAFLD. Liver biopsy remains an essential diagnostic tool when AIH cannot be excluded, and immunosuppressive treatment is being considered. Histological examination of liver tissue can reveal various patterns, including necroinflammatory, cholestatic, and steatotic changes, as well as vascular injury and cytoplasmic alterations [46]. Despite its utility, liver histology has limited value in the differential diagnosis, particularly in the early stages of AIH, due to overlapping histological features. For instance, common findings like portal lymphoplasmacytic infiltration and interface hepatitis are present in both DI- ALH and AIH. While advanced hepatic fibrosis generally suggests AIH, none of the histological features can definitively differentiate DI-ALH from AIH [47,48]. In our study, liver biopsy was performed exclusively in patients with clinically significant liver injury that required hospitalization. However, the biopsy results did not reveal any pathognomonic features that could clearly categorize the patients as suffering from DI- ALH or AIH.

Among the various causality assessment methods for diagnosing DILI, RUCAM remains the most widely used in clinical research globally [38]. While it is a valuable tool, it should be emphasized that RUCAM alone may be insufficient due to its limited reliability and potential documentation gaps [49]. Despite these limitations, we, in alignment with other studies, opted to utilize this method alongside the Simplified AIH criteria for excluding AIH. Although these criteria demonstrate moderate diagnostic sensitivity, they are particularly useful in guiding treatment decisions, especially when a score of 6 or higher is obtained in suspected AIH cases [17]. In our study, with the exception of case 13, none of the participants met the diagnostic criteria for AIH.

According to the European Association for the Study of the Liver (EASL) Clinical Practice Guidelines for DILI, a positive rechallenge is regarded as the most definitive evidence for establishing drug causality in suspected DILI cases. It is specifically defined as an increase in ALT levels exceeding three times the ULN following re-administration of the suspected drug, provided that baseline ALT levels were within the normal range prior to rechallenge [50,51,52]. Despite its diagnostic value, rechallenge is infrequently conducted due to the associated risk of serious adverse outcomes, with reported mortality rates reaching as high as 13% in certain instances [50]. In our study cohort, none of the patients underwent intentional rechallenge.

Consistent with the preponderance of available data, our findings strongly suggest that liver injury following IVMP pulses aligns more closely with DI-ALH rather than classical AIH. Notably, in cases without GC administration, particularly those with mild, non-clinically significant liver injury, spontaneous normalization of liver enzymes occurred without specific intervention. By contrast, with the exception of case 13, two patients with clinically significant liver injury (cases 2 and 3) who received GC therapy achieved full resolution, with no recurrence when treatment was gradually tapered. Patient 3 represents an intriguing case, having experienced a prior episode of clinically significant liver injury that resolved spontaneously, with liver biochemistry remaining normal for five years despite the absence of targeted therapy.

On the other hand, the possibility of latent AIH flares cannot be entirely excluded, although supporting evidence remains limited. Even in studies favoring the DILI hypothesis, such as the work by Nociti et al., three of six severe cases were still classified as AIH [20]. Another notable example is the study by Cação et al., which reported three cases of liver injury in MS patients following steroid pulse therapy, all diagnosed as AIH. However, this study has significant limitations: the diagnostic criteria for AIH were not specified, immunological tests were normal in all cases, and liver biopsy findings uniformly revealed lymphoplasmacytic infiltration. Interestingly, one case resolved spontaneously without treatment [53]. Similarly, Rigopoulou et al. identified AIH as a common diagnosis in MS patients with abnormal liver function tests, frequently associated with immunomodulatory treatments. In these cases, diagnosis was confirmed through laboratory findings and liver histology, with most patients responding well to prednisolone and immunosuppressive agents such as MMF [54].

The differential diagnosis between DI-ALH and AIH is particularly valuable, as it can significantly influence subsequent therapeutic decisions. Specifically, DI-ALH often resolves spontaneously upon withdrawal of the offending agent, whereas AIH typically necessitates prolonged immunosuppressive therapy, with a considerable risk of relapse if treatment is prematurely discontinued [55]. Therefore, in cases of confirmed AIH, immunosuppressive therapy is clearly warranted [56]. In the remaining cases, management remains contentious due to the lack of established guidelines. Based on the current evidence, the therapeutic approach should be tailored to the severity of the liver damage and individualized for each case. For asymptomatic mild to moderate liver injury, close monitoring without immediate treatment is a reasonable strategy. If liver biochemistry deteriorates during follow-up or TBIL levels remain elevated despite normal ALT levels, GCs should be administered [57]. GCs are central to managing clinically significant liver injury, even amidst ongoing debate regarding their use. In severe DI-ALH, GCs can improve liver function without significantly increasing mortality risk and can be safely discontinued once remission is achieved [24,58]. While evidence on the optimal steroid regimen for DI-ALH is limited, expert guidelines recommend an initial dose of 40 mg prednisolone tapered over 1–3 months, with discontinuation within 3–6 months [38]. Our center follows this approach, with case 11 responding well to budesonide, indicating that other steroid therapies may also be effective.

A key point of convergence among various studies is the generally favorable prognosis of liver injury following IVMP pulses. Research consistently highlights the safety and benefits of GC therapy, particularly its association with faster normalization of liver biochemistry, a crucial factor for MS patients receiving DMTs with potential hepatotoxic risks [45]. In our study, GC treatment resulted in a significantly faster biochemical normalization, with a mean recovery period of 48 days compared to 60.71 days in non-GC-treated individuals. Interestingly, a rapid biochemical response to GC therapy has also been proposed as a diagnostic tool for distinguishing DI-ALH from classical AIH, potentially offering a more reliable indicator than histological features alone [15].

However, resistance to standard GC treatment may occur in a subset of patients. For example, in the study by Allgeier et al., one patient required liver transplantation [15]. Similarly, in our cohort, case 13, who had a prior history of AIH, failed to respond to MP and required salvage therapy with rituximab, raising questions about the reliability of MP in such cases. Although fatal outcomes are rare, isolated case reports in the literature have documented them [25].

Follow-up recommendations for MS patients receiving IVMP pulses suggest liver biochemistry monitoring before infusion and again two weeks afterward. For at-risk individuals, more frequent monitoring is advised: weekly during treatment and monthly for the following year [35]. Recently, the DILI consortium and the International Autoimmune Hepatitis Group (IAIHG) recommended that patients with DI-ALH should undergo an initial follow-up every 2–4 weeks, followed by evaluations at 1, 3, 6, 12, 18, and 24 months. Subsequently, annual follow-up is advised for a total duration of five years. These guidelines are based on Spanish and Latin American registry data showing a 50% relapse rate over four years [59]. In contrast, our study observed no recurrences over a mean follow-up period of 4.30 years, with most cases (10/13) remaining free of liver biochemistry abnormalities throughout the 5-year follow-up. However, it is important to acknowledge that in the three remaining cases, which are presented with clinically significant liver damage, the follow-up period was limited to two years, the time elapsed since their initial diagnosis. Therefore, we recommend this algorithm, except for patients at increased risk of developing hepatitis, for whom the initial follow-up should be scheduled one week after IVMP treatment.

For future exacerbations, two main treatment strategies are available: either administering the standard IVMP regimen followed by a gradual tapering of prednisolone over 1–2 months with close monitoring or considering alternative steroids such as dexamethasone. At our center, we prefer the first approach, as experience with dexamethasone remains limited [35,36,60]. Nevertheless, dexamethasone can be a viable alternative, although specific dosage regimens cannot be clearly defined. As previously mentioned, individual case reports from the literature have shown that administering dexamethasone in doses ranging from 50 to 92 mg per day for three consecutive days was both effective and safe [35,36].

**Figure 2:**
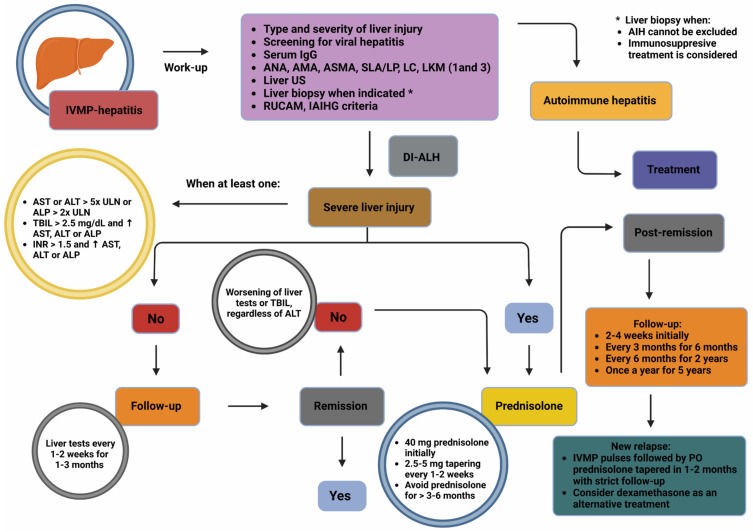
Schematic algorithm depicting the proposed diagnostic and treatment approach for liver injury following IVMP pulse administration. Abbreviations: AIH: autoimmune hepatitis; ALP: alkaline phosphatase; ALT: alanine aminotransferase; AMA: anti-mitochondrial antibodies; ANA: antinuclear antibodies; ASMA: anti-smooth muscle antibodies; AST: aspartate aminotransferase; DI-ALH: drug-induced autoimmune-like hepatitis; IAIHG: International Autoimmune Hepatitis Group; IgG: immunoglobulin G; INR: international normalized ratio; IVMP: intravenous methylprednisolone; LC: anti-liver cytosol antibodies; LKM: liver–kidney microsomal antibodies; MS: multiple sclerosis; PO: peros; RUCAM: Roussel Uclaf Causality Assessment Method; SLA/LP: soluble liver antigen/liver–pancreas antigen complex; TBIL: total bilirubin; ULN: upper limit of normal; US: ultrasound. Created in BioRender. Kounatidis, D. (2025) https://BioRender.com/v51w817, accessed on 1 March 2025.

A schematic algorithm for diagnosing and treating liver injury from IVMP in MS patients is shown in Figure 2, and is applicable to those with IVMP-induced hepatitis in other autoimmune diseases.

The primary limitations of our study include the observational nature of the study, the small sample size, the exclusive inclusion of Caucasian patients, and the fact that liver biopsies were only performed in patients with severe liver injury, leaving most participants without histopathological confirmation. Despite these limitations, the study possesses notable strengths. It features a comprehensive initial evaluation of patients, consistent measurement of immunological parameters by a single laboratory, and close follow-up, initially over six months for all patients, and subsequently for up to five years for the majority. However, it is important to note that patients with severe liver injury were followed for only two years, since, as previously discussed, these cases were diagnosed more recently. Crucially, the study demonstrates the beneficial and safe role of GC therapy when indicated. Additionally, the diagnostic and therapeutic algorithm derived from our findings, in conjunction with the existing literature, provides substantial guidance for clinicians managing similar cases and lays the foundation for larger-scale studies in the future.

## 5. Conclusions

Liver injury has emerged as a notable side effect of IVMP pulses in patients with autoimmune diseases, including those with MS, necessitating regular monitoring of liver function. Differentiating DI-ALH from idiopathic AIH remains challenging due to their overlapping clinical and biochemical features. Our study supports the diagnosis of DI-ALH, demonstrating favorable outcomes without relapsing following steroid withdrawal in most patients. The majority of affected individuals were women with a mean age of approximately 40 years, a pattern in line with MS demographics. Liver damage manifested two weeks after IVMP withdrawal, consistently displaying a hepatocellular motif, while clinical symptoms were absent, particularly in non-severe cases. Positive ANA were detected in more than half of the subjects, reflecting the autoimmune nature of the condition, and approximately 23% of patients required hospitalization. Although liver biopsy, when performed, was insufficient on its own to reliably distinguish DI-ALH from idiopathic AIH, it remains an essential diagnostic tool in cases where AIH cannot be excluded. Liver biopsy is particularly recommended for patients in whom GC therapy is being considered, with prednisolone being the preferred treatment option. Mild to moderate liver injury often resolves spontaneously, while severe cases usually benefit from GC therapy, which accelerates liver biochemistry normalization. Steroids can be safe and effective, but treatment must be individualized, with long-term monitoring to detect and manage potential relapses. The findings of our study align with the majority of existing research, which predominantly comprises observational studies. These collective results underscore the need for large, well-designed clinical trials to provide more robust evidence.

## Figures and Tables

**Table 1:** Main demographic, clinical, and laboratory characteristics of the patient population.

Parameter	Findings
Female gender	12/13
Median age	40.07 (25–60)
On DMT/other treatment	6/13
Normal LFTs pro-IVMP pulse	13/13
Previous IVMP pulse	6/13
Median latency period	37.46 (15–75)
Presence of symptoms	3/13
Mean ALT (U/L) on admission	539.61 (93–2318)
Mean peak ALT (U/L) on follow-up	618.46 (97–2318)
Mean TBIL (mg/dL) on admission	2.54 (0.46–11.60)
Serum IgG > 1600 mg/dL on admission	2/13
Positive ANA on admission	8/13
Hepatocellular liver injury	13/13
Severe liver injury/Hospitalization	3/13
Mean RUCAM	7 (5–10)
Simplified AIH score	1/13 (probable > 6)
Treatment with GCs	6/13
Responsive to GCs	5/6
Type of GC used	Prednisolone (4/6) Budesonide (1/6) Methylprednisolone (1/6)
Cases with remission	12/13
Remission (days)	55.41 (14–100)
Remission (days)without GC treatment	60.71 (30–90)
Remission (days) after GC treatment	48 (14–100)
Mean follow-up period (years)	4.30 (2–5)

Abbreviations: AIH: autoimmune hepatitis; ALT: alanine aminotransferase; ANA: antinuclear antibodies; DMT: disease-modifying therapy; GC: glucocorticoid; IgG: immunoglobulin G; IVMP: intravenous methylprednisolone; LFT: liver function test; RUCAM: Roussel Uclaf Causality Assessment Method; TBIL: total bilirubin; ULN: upper limit of normal.

## Data Availability

The original contributions presented in this study are included in the article/Supplementary Material. Further inquiries can be directed to the corresponding author.

## Supplementary Materials

The following supporting information can be downloaded at: https://www.mdpi.com/article/10.3390/biom15030437/s1, Table S1: Specific characteristics of the patient cohort; Table S2: Key Findings From Initial Work-Up of the Patient Cohort.

## Abbreviations

AIDS: acquired immunodeficiency syndrome; AIH: autoimmune hepatitis; ALP: alkaline phosphatase; ALT: alanine aminotransferase; AMA: anti-mitochondrial antibodies; ANA: antinuclear antibodies; ASMA: anti-smooth muscle antibodies; AST: aspartate aminotransferase; CMV: Cytomegalovirus; CNS: central nervous system; DI-ALH: drug-induced autoimmune-like hepatitis; DILI: drug-induced liver injury; DMT: disease-modifying therapy; EASL: European Association for the Study of the Liver; EBV: Epstein–Barr virus; GC: glucocorticoid; HAART: highly active antiretroviral therapy; HIV: human immunodeficiency virus; HLA: human leukocyte antigen; IAIHG: International Autoimmune Hepatitis Group; IFN-γ: interferon-gamma; IgG: immunoglobulin G; IL: interleukin; IRIS: immune reconstitution inflammatory syndrome; IVMP: intravenous methylprednisolone; LC: liver cytosol antibodies; LFT: liver function test; LKM: liver–kidney microsomal antibodies; MAFLD: metabolic-associated fatty liver disease; MMF: mycophenolate mofetil; MP: methylprednisolone; MS: multiple sclerosis; PO: peros; RUCAM: Roussel Uclaf Causality Assessment Method; SLA/LP: soluble liver antigen/liver–pancreas complex; TBIL: total bilirubin; TNF-α: tumor necrosis factor-alpha; ULN: upper limit of normal; US: ultrasound.
