# Supplementary material for: Liver Injury Following Intravenous Methylprednisolone Pulse Therapy in Multiple Sclerosis: The Experience from a Single Academic Liver Center
Source: Biomolecules. 2025 Mar 19;15(3):437. doi: 10.3390/biom15030437 (PMC11940579; doi:10.3390/biom15030437)
Supplement: Supplementary file 1 [file biomolecules-15-00437-s001.zip › biomolecules-3456705-supplementary.pdf]

Case	Gender/ Age	DMT/other treatment	Normal LFTs pro-IVMP	Previous IVMP	MP regimen	Latency period	Symptoms	Type of liver injury	Inpatient care	Treatment	Remission (days)
**1**	F/37	No	Yes	No	1 g daily, 3 days	30	No	Hepatocellular	No	Prednisolone	14
**2**	F/52	IFN-beta	Yes	No	1 g daily, 3 days	60	Nausea, jaundice	Hepatocellular	Yes	Prednisolone	100
**3**	F/48	No	Yes	Yes	1 g daily, 2 days	25	Fever, nausea, diarrhea	Hepatocellular	Yes	Prednisolone	50
**4**	F/29	No	Yes	Yes	1 g daily, 5 days followed by MP for 4 days	37	No	Hepatocellular	No	No	80
**5**	F/40	No	Yes	No	1 g daily, 5 days	75	No	Hepatocellular	No	No	30
**6**	F/46	No	Yes	No	1 g daily, 5 days	53	No	Hepatocellular	No	No	62
**7**	F/60	No	Yes	Yes	1 g daily, 3 days	35	No	Hepatocellular	No	No	45
**8**	F/32	No	Yes	No	1 g daily, 5 days	15	No	Hepatocellular	No	No	40
**9**	F/25	Glatiramer acetate	Yes	Yes	1 g daily, 5 days	40	No	Hepatocellular	No	No	78
**10**	M/38	IFN-beta	Yes	No	1 g daily, 3 days	20	No	Hepatocellular	No	Prednisolone	62
**11**	F/41	Glatiramer acetate	Yes	Yes	1 g daily, 3 days followed by PSL for 12 days	52	No	Hepatocellular	No	Budesonide	14
**12**	F/39	Ofatumumab	Yes	Yes	1 g daily, 3 days	30	No	Hepatocellular	No	No	90
**13**	F/34	MMF	Yes	No	1 g daily, 3 days	15	Jaundice	Hepatocellular	Yes	MP	No

Table S1: Specific characteristics of the patient cohort. Abbreviations: DMT: disease-modifying Therapy; F: female; IFN-beta: interferon-beta; IVMP: intravenous methylprednisolone; LFTs: liver function tests; MMF: mycophenolate mofetil; MP: methylprednisolone.

Case	AST/ALT (U/L)	ALP/GGT (U/L)	Peak ALT (U/L)	TBIL (mg/dL)	INR	IgG (mg/dL)	Antibodies	Liver Ultrasound	Liver biopsy	RUCAM	AIH score
**1**	60/112	55/12	206	0.46	0.7	1380	ANA (1:160)	Normal	No	7	< 6
**2**	2160/2318	217/194	2318	11.60	1.7	1530	No	Normal	Portal inflammation with neutrophils/lymphocytes infiltration	6	< 6
**3**	212/474	120/726	542	7.40	1.4	1894	ANA (1:320)	Normal	Denied	10	< 6
**4**	202/544	64/66	544	1.5	0.9	697	ANA (1:80)	Normal	No	8	< 6
**5**	56/93	55/16	120	0.68	0.8	1134	No	Fatty liver	No	8	< 6
**6**	104/261	49/17	489	0.80	0.9	820	ANA (1:160)	Normal	No	7	< 6
**7**	61/97	62/68	97	0.72	0.8	1364	No	Normal	No	7	< 6
**8**	92/211	45/34	211	0.69	0.7	856	No	Normal	No	8	< 6
**9**	522/1115	40/44	1115	1.2	1.1	1255	ANA (1:80)	Normal	No	6	< 6
**10**	109/208	73/32	208	0.77	0.9	986	ANA (1:320)	Normal	No	7	< 6
**11**	96/152	59/14	389	0.80	1.1	1104	ANA (1:640)	Normal	No	6	< 6
**12**	88/169	52/28	301	0.74	0.9	1185	No	Normal	No	6	< 6
**13**	1028/1261	137/336	1500	5.70	1.52	2000	ANA (1:80) ASMA (1:80) LKM (1:40)	Normal	Acute portal hepatitis with centrilobular necrosis	5	6 (probable)

Table S2:. Key Findings From Initial Work-Up of the Patient Cohort. Abbreviations: AIH: autoimmune hepatitis; ALT: alanine aminotransferase; ANA: antinuclear antibodies; ALP: alkaline phosphatase; ASMA: anti-smooth muscle antibodies; AST: aspartate aminotransferase; GGT: gamma-glutamyl transferase; IgG: immunoglobulin G; INR: International Normalized Ratio; LKM: liver-kidney microsomal antibodies; RUCAM: Roussel Uclaf Causality Assessment Method; TBIL: Total Bilirubin.
